# Plasticity in perception: insights from color vision deficiencies

**DOI:** 10.12703/b/9-8

**Published:** 2020-11-13

**Authors:** Zoey J Isherwood, Daniel S Joyce, Mohana Kuppuswamy Parthasarathy, Michael A Webster

**Affiliations:** 1 Department of Psychology, University of Nevada, Reno, NV, USA

**Keywords:** Neural plasticity, adaptation, vision, color vision, color deficiencies

## Abstract

Inherited color vision deficiencies typically result from a loss or alteration of the visual photopigments absorbing light and thus impact the very first step of seeing. There is growing interest in how subsequent steps in the visual pathway might be calibrated to compensate for the altered receptor signals, with the possibility that color coding and color percepts might be less severely impacted than the receptor differences predict. These compensatory adjustments provide important insights into general questions about sensory plasticity and the sensory and cognitive processes underlying how we experience color.

## Introduction

Plasticity is a hallmark of sensory processing. Environments vary widely over space and time, and sensory systems must continuously recalibrate to optimize coding for the current context^1,2^. Importantly, the sensory apparatus also varies widely among individuals, and even within an individual there are dramatic changes over time (e.g. development and aging) and space (e.g. between central and peripheral vision). Plasticity must therefore correct for the properties not only of the world but also of the observer. These adjustments can arise at many structural levels, from large-scale cortical reorganization (e.g. when cortical areas normally devoted to one sense are recruited by other modalities when that sense is lost)^3^ to local synaptic dynamics^4^ and sensitivity regulation within cells^5–7^ and networks^8,9^. They can also occur over many timescales^10^, from rapid response changes within milliseconds^11^ to experience-dependent development^12,13^. Finally, they occur throughout the processing hierarchy, from adjusting receptor sensitivity to tuning cognition and learning. A major unresolved issue in neuroscience is defining the actual number of distinct mechanisms underlying neural plasticity, how they are manifest, and how they interact.

Here we discuss general issues and insights about plasticity within the specific context of common inherited color vision deficiencies. Recent studies have highlighted the potential for compensating for a color vision deficiency so that the world is described as richer in color than the sensory losses would predict. However, the nature of these compensations and whether they reflect perceptual or conceptual adjustments^14^ remain poorly understood. For example, a perceptual adaptation could reflect changes in the sensory signals encoding color, while a conceptual adjustment might be learning to label the percept with the same color name that others use^15–18^. Exploring plasticity through the lens of color vision provides a rich and unique opportunity for revealing how and how far neural coding and perceptual experience can be calibrated to discount natural physiological variations within and between observers and one that can also provide new insights into longstanding questions and controversies about the processes mediating human color vision.

## Human color vision

**Figure 1.**
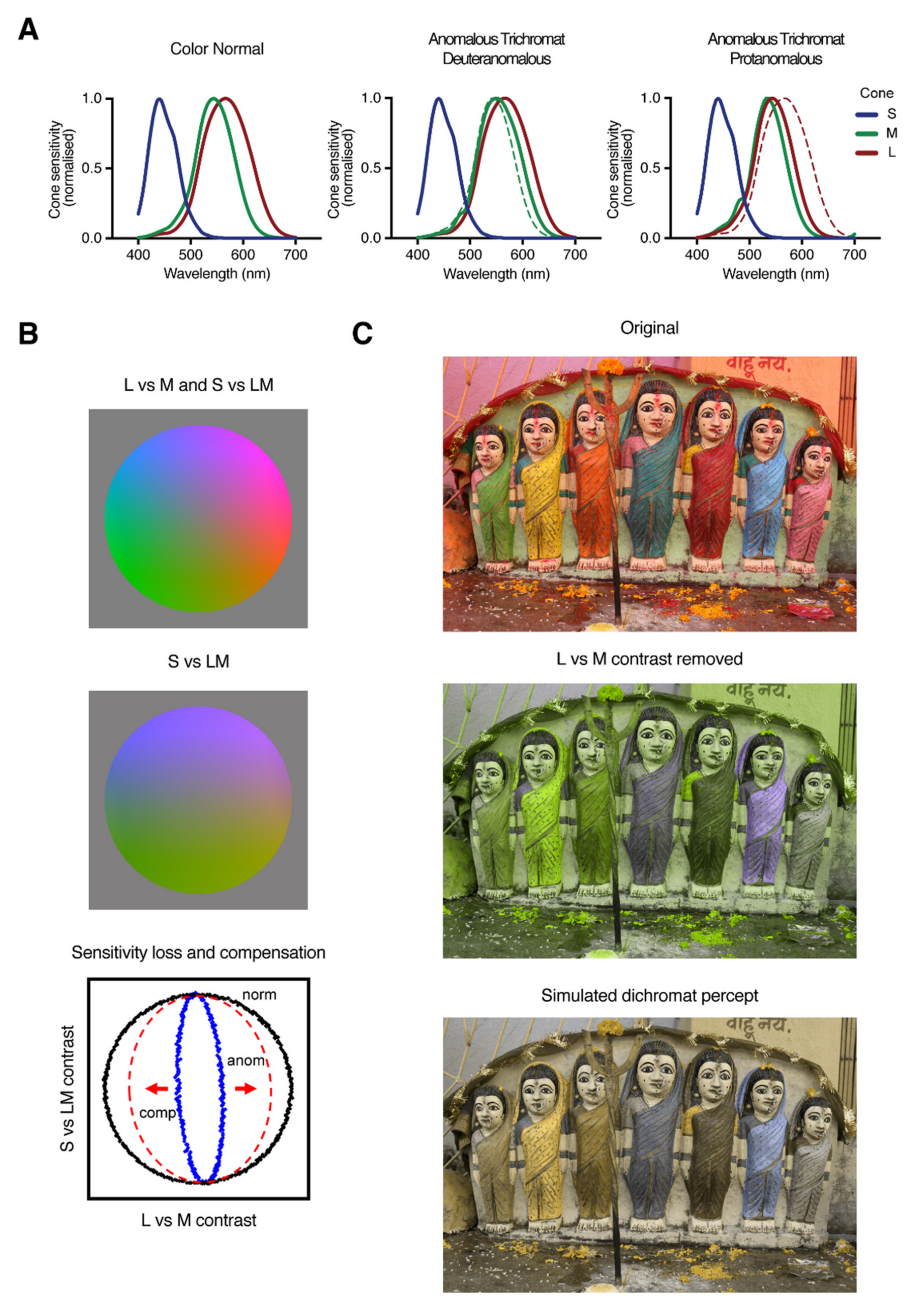
Simulations of color vision deficiencies. **A**. Routine trichromatic color vision is based on three classes of receptors with different but overlapping sensitivities. Most inherited deficiencies affect the L (protan) or M (deutan) cones, leading to their loss (dichromacy) or a shifted peak toward the unaffected cone (anomalous trichromacy). **B**. A space defined by the color differences signaled by comparing L vs. M cones or the S vs. LM. Reducing the L-M signal collapses the color space toward the S vs. LM axis but could be compensated by a post-receptoral amplification. **C**. Filtering an image to remove the L-M contrasts removes the distinctions between reddish and greenish colors. Reports from unilateral dichromats suggest they may experience the residual colors as blue-yellow variations^38^.

Most animal species have the capacity for color vison, and in all known cases it is based on detecting light with two or more photoreceptor classes that differ in the wavelength sensitivity of their photopigments^19–22^. However, having more types of receptors does not necessarily confer a higher dimensionality of color vision^23^. Most humans have three classes of cone receptors maximally sensitive to short (S), medium (M), or long (L) wavelengths, and thus normal (or, more aptly, routine) color vision is trichromatic. Encoding color further depends on the neural machinery for comparing the relative cone responses, for example to determine whether the L cones or M cones are more excited by a light spectrum. These comparisons begin in the retina, in post-receptoral neurons that receive inputs of the same or opposite sign from different receptor types, and are carried within three “cardinal” mechanisms^24^ with distinct cell types and pathways, named for their projections to different layers of the lateral geniculate nucleus^25–28^. Cells in the magnocellular (M) pathway sum the L and M cones’ signals and are the substrate of our luminance sensitivity (L+M)^29^. Chromatic information is instead carried by two cone-opponent cell types that receive opposing signals from the L and M cones (L-M, the parvocellular or P pathway) or from S cones opposed by both L and M (S-LM, the koniocellular or K pathway). Figure 1 illustrates the colors as defined by these two chromatic dimensions. However, these mechanisms describe only the initial steps of color coding. There are major further transformations of the cone-opponent signals in the cortex, and different transformations may arise at several different cortical stages^30–32^. Moreover, even within the retina, there is a possibility that color percepts are carried within pathways that combine the cones in different ways than the cardinal mechanisms^33,34^.

## Inherited color vision deficiencies

Inherited color vision deficiencies primarily reflect alterations in the genes coding the cone photopigment opsins. The genes coding the L and M opsins lie in tandem on the X chromosome^35^. Errors in the L or M genes can lead to a loss of one receptor class (dichromacy) or to shifts in the spectral sensitivity (anomalous trichromacy) (Figure 1). The latter reduces the spectral separation between the L and M cones and thus reduces the difference signals (L-M) conveying chromatic information, resulting in reduced discrimination for this chromatic dimension. As an X-linked recessive trait, color vision deficiencies of this type are the most common and affect about 6% of males but are rare in females. Instead, female carriers for color vision deficiencies (with a normal gene on one X chromosome and modified gene on the other) might express both to develop a fourth dimension for color^36^. However, whether and when this leads to functional tetrachromacy has been challenging to resolve^37^.

Simulations of color vision deficiencies are common and work by filtering the image to remove or alter the chromatic information that should be lost or weakened by their altered sensors (Figure 1). These illustrations are often closer to depicting what a trichromat would experience if they suddenly lost one class of receptors but have also incorporated comparisons from rare individuals with a color loss in only one eye to better simulate a dichromat’s experience^38^. However, few simulations have tried to capture the impact of compensation for the receptor losses^39^.

Characterizing compensatory processes for inherited color vision deficiencies provides an ideal natural experiment for exploring the types and limits of plasticity in neural processing and perception. First, the deficit arises from only a single discrete change in the visual system that is restricted to the first step of vision, when the light is captured by the receptor^35^. This allows characterizing how the rest of the system can reorganize given a simple and highly stable change in its inputs. Second, each individual has had a lifetime experiencing the world through their receptor complement. Thus, their perceptual capacities can reveal adaptations over timescales that are orders of magnitude longer than are typically studied in a lab, potentially revealing different mechanisms as well as the ultimate limits of sensory plasticity. Third, the spectral sensitivity differences between observers can be very precisely quantified, allowing precise predictions about their color perception and sensitive tests for any adaptive changes. Finally, a wide variety of techniques have been developed to probe color vision and to try to isolate different stages of processing, from the photopigments and early sensory coding to cognition and consciousness. Thus, different measurements can be deployed to target different levels or properties of the system in order to assess at what stages or in what tasks a compensation for the color vision deficiency occurs and what this implies about the underlying mechanisms.

## Compensation for color vision deficiencies

Because dichromats completely lose one cone class, in principle there is no signal that the visual system could restore. Yet dichromats can reliably use a wide range of color terms that map onto color naming patterns for trichromats, and these abilities have been attributed to both sensory processing and learning how colors are communicated^14,40–42^. Dichromats also can become trichromatic for large fields by taking advantage of the changes in spectral sensitivity across the retina or information provided by rod receptors^43^. The rods are a fourth receptor class specialized for sensing under dim light levels. The rod system is typically described as “color blind”, yet there are many examples of rod contributions to color vision^44^ and color can be experienced even at low light levels at which only rods are active, perhaps because of learned associations with cone-mediated percepts^45^. Another intriguing possibility is the recruitment of signals arising from the intrinsically photosensitive retinal ganglion cells (ipRGCs). These recently discovered cells are in the output neural layer of the eye but directly absorb light using melanopsin as the photopigment^46,47^. The ipRGC pathway may primarily serve non-image forming, or non-perceptual, visual function, including regulating circadian rhythms, sleep, mood, and cognition^48^. However, ipRGCs also receive inputs from cones and rods and project to visual pathways^49,50^. Their contributions to color perception remain difficult to isolate, but excitation of this pathway has been found to influence both brightness and color percepts^51–55^. To our knowledge, the possible role of these signals in color vision deficiencies remains unexplored. However, dichromats may offer a more powerful test case for probing color coding and ipRGCs because these observers may be more dependent on their signals and also because the signals themselves may be easier to isolate from the cone responses.

Recently, several theoretical and empirical studies have also explored compensation for anomalous trichromacy^56^. In this case, the smaller differences in the L and M cone sensitivities could in principle be discounted simply by amplifying the gain in the post-receptoral neurons that compare these signals^39,57^. As noted, this comparison is initially carried out in cone-opponent cells that receive opposite inputs from the L and M cones and is carried through the parvocellular pathway^25,26^. The rationale for amplifying their responses when the inputs are weaker is grounded in information theory, which predicts that each cell should center and scale its limited operating range for the distribution of inputs in order to maximize the information it can transmit^58–61^. Rescaling responses to the magnitude of luminance or chromatic variation (or contrast) is a well-established form of visual adaptation documented in countless behavioral and physiological studies^6,7,62,63^. Typically, these involve stimulating the system with high contrast and measuring the resulting sensitivity losses. Yet a small number of studies have also shown that when observers are exposed to weaker-than-normal luminance contrasts, the responses instead increase^64–66^, suggesting that perceived saturation should also increase in an observer habitually exposed to low L-M contrasts.

There are a number of unsolved puzzles about the impact of such post-receptoral gain adjustments. For example, adaptation could amplify not only the signal but also the noise, and thus the consequences for perception depend on where in the system sensitivity regulation and the limiting noise occur^39,58,67^. In addition, while color contrast adaptation is readily observed in the visual cortex, color-opponent cells at the level of the retina and LGN show substantially less adaptation^68,69^ (but see 70). This raises the possibility that the system could restore optimal coding in the cortex even though the chromatic signals in the retina and LGN remain weak and thus poorly calibrated to the natural environment. One potential rationale for this is that P cells encode both color and lightness information (because the weights of the L and M cone inputs vary substantially^25^ and because the cone inputs are segregated into different spatial subregions of the cell’s receptive field^71^). This means that gain changes at this level not only would amplify the weakened chromatic signal but also could “over-amplify” the better preserved lightness signals. In the cortex, lightness and chromatic information may become decoupled^72,73^, and it may therefore be more advantageous to adapt at this stage in order to separately calibrate these signals. A still further possibility is that the retina and LGN can adjust in different ways or over longer timescales than are revealed by short-term adaptation experiments. Adaptation to color can occur over many timescales^74–77^. However, it remains uncertain whether adjustments with different time constants reflect similar processes and consequences but tracking different rates of change or if they reflect fundamentally different mechanisms that calibrate different aspects of the system.

Empirically, the primary evidence for compensation has come from studies comparing the perceptual reports of anomalous trichromats. For example, in a perceptual grouping task, some anomalous observers perceived L-M color differences as more salient than expected^78^. Similarly, estimates of the color responses from multidimensional scaling^79,80^ or contrast scaling^81^ have found that the L-M percepts are stronger than predicted from the observer’s detection thresholds and can come close to the responses for routine trichromatic observers.

However, here again there are many unresolved questions. First, these studies rely on observers’ subjective reports, and thus it is difficult to infer from these reports what they actually “see.” For example, individuals could learn to interpret or label the same stimuli with the same scale or terms that others use even if they perceive them very differently, and they might base their reports on very different stimulus cues^14,41,82^. As noted, even dichromats can exhibit nearly normal patterns of basic color naming but might rely on different cues for this, such as luminance variations or rod signals. As such, some putative measures of color appearance and compensation may reflect more cognitive stages (such as how the sensory signals are interpreted or categorized) rather than the strength of the sensory signals themselves. Objective measures of the chromatic contrast responses in anomalous trichromacy remain lacking. However, recent studies have begun to probe compensation for color vision deficiencies with neuroimaging. For example, an fMRI study^83^ found that the BOLD response to L-M contrast was amplified within but not before early visual cortical areas, implicating V1 or V2 as the earliest site of the gain compensation. Another study using visual evoked potentials (VEPs) also found amplified signals, but only when the stimuli were viewed binocularly, again implicating a cortical locus for the response gains^84^. For monocular viewing, VEP amplitudes are substantially reduced and diagnostic of the deficiency^85^.

A second question is under what conditions the compensation is manifest. Clearly, the sensitivity losses are not completely discounted because anomalous trichromats fail color screening tests, which measure the ability to discriminate small color differences. Similarly, the simple loss hypothesis closely predicts their color matching behavior (i.e. when two lights with different physical spectra will look the same), and the color matches of dichromats have played a central role in deriving the cone spectral sensitivities^86^. The presumed amplification instead occurs above the detection threshold, when the system “knows” a signal is there. A related example is the phenomenon of contrast constancy. Thresholds for detecting spatial contrast (e.g. in spatial sinewave gratings) are lowest for medium spatial scales, forming the characteristic bandpass contrast sensitivity function. However, when the gratings are slightly above threshold, the perceived contrast is nearly independent of spatial frequency^87,88^. With regard to color, greater constancy or compensation may also be manifest when the task reflects cognitive factors (e.g. learning color categories) rather than early sensory signals^14^.

A further challenge for interpreting these studies is that anomalous trichromacy is itself a very heterogeneous condition^56^. Observers can vary widely in the separation of their longer wave cones, as well as which cone class is altered, and this separation does not always predict even their performance at threshold^89,90^. For example, some anomalous observers who are revealed by their altered color matches nevertheless have fine color discrimination. How these individual differences impact the form and extent of compensatory adjustments remains an important question to address.

Finally, it should be emphasized that even in measures of color appearance or salience, the degree of compensation is rarely complete. That is, suprathreshold percepts may be amplified relative to threshold sensitivity but, in most anomalous observers, still fall short of the L-M responses of routine trichromats^78,79,81,91^. Understanding why compensation is incomplete is as important as understanding why it occurs at all. In the case of color, there may again be many factors limiting plasticity, from the problems of amplifying noise to the possibility that anomalous observers are basing their judgments on different cues (e.g. lightness variations) that may be inherently weaker. Another potential limit is that the L and M cones are thought to differ only in their opsins and thus do not have a unique physiological signature. The visual system must therefore learn the identity of each cone from the patterns of their responses to the spectra the observer experiences^92,93^. Misclassifications are more likely the smaller the differences between these responses, and this could introduce errors in post-receptoral coding that cannot be undone by simply amplifying those differences.

## Restoring color vision

We have emphasized the advantage of assessing plasticity when the adjustment is to a stable and permanent property of the visual system. But what if that property suddenly changes? There is now the prospect for viral vector-mediated gene therapy to introduce the missing photopigment in the retina^33^. Dichromatic adult monkeys whose eyes were injected with a missing L pigment gene expressed the pigment over time and became trichromatic in their color discriminations^94^. This suggests that even in the adult, the post-receptoral machinery can exploit the added input signals, though the extent to which this involves tapping into existing pathways versus recalibration remains uncertain. Beyond the expanded capacity to detect colors, it is also unknown how the added receptor type will impact the perceptual experience of color^95^ and what forms of plasticity might adjust for these.

Techniques have also been developed to optically enhance reddish-greenish contrasts for color-anomalous observers by using notch filters. These cannot restore normal color vision^96^ but can alter the relative salience of different colors. A recent study found that after wearing these glasses for just a few days, the participants reported experiencing stronger L-M contrasts, even with the glasses removed^97^. This is surprising given that the observers had a lifetime to adjust to their cone sensitivities and because the changes are in the opposite direction to the effects predicted by adaptation (since adapting to stronger colors should have resulted in reduced sensitivity). However, similar effects have been found in perceptual training for other visual deficits (e.g. amblyopia)^98–100^, where requiring the observer to attend to the weakened visual signals allows them to process them more effectively. An implication of such results is that the visual system is capable of even greater plasticity when the stimulus or task demands it, and these effects have been widely studied in the context of perceptual learning^101–104^. How this learning interacts with other forms of plasticity like adaptation remains poorly understood but is being actively investigated^105–109^.

## Implications for routine trichromatic color vision

Importantly, the processes compensating for a color vision deficiency are also at play in calibrating the sensitivity and perception of individuals with routine trichromacy. For example, many authors have noted that because of the spectral overlap in the L and M cones (and, to a lesser extent, the S cones), their responses are very similar and for natural spectra are very highly correlated^110–112^. This means that the L-M chromatic signal is many times weaker than the L+M signal, which underlies our luminance sensitivity. Based on these cone contrasts, the world should appear to vary much more in brightness than color. However, post-receptoral sensitivity is correspondingly much higher for the chromatic cone contrasts^57,113^, to an extent that roughly matches the gamut of color signals we are exposed to in our environment^114^. From this perspective, then, the gain adjustments in the anomalous observers are simply the same adjustments that operate in every observer.

A further important point is that color vision deficiencies are just a more extreme case of the enormous variability inherent in human color vision. Individuals who are all classified as “normal” trichromats nevertheless differ widely in their spectral sensitivities because of differences in the lens and macular screening pigments, the specific absorption spectra of the cones, and the relative number of the L and M cones^115–117^. Spectral sensitivity differences influence an observer’s color matches (i.e. which physically different spectra appear identical) but have surprisingly little impact on measurements of color appearance^118,119^. For example, what looks gray does not vary with age despite the yellowing lens^120,121^ and does not vary with space despite the drop in macular screening pigment outside the central fovea^122,123^, and the relative sensitivities of the different cone classes also show little change with age^124,125^. Similarly, the L to M cone ratios strongly impact luminance sensitivity but have little influence on color percepts^126–128^. Again, this is because each individual’s visual system is calibrated for their environment^63,118^. Thus, what looks gray is as much a property of the environment (e.g. the average spectral stimulus) as the visual system representing it^129,130^.

Comparing different stages of color coding in routine and color-deficient observers could continue to help resolve some of the ongoing challenges of understanding the bases for human color vision and what different measurements of color perception actually reflect. Traditional models of color vision assume that subjective color appearance depends on two chromatic opponent processes signaling red vs. green or blue vs. yellow sensations. Yet, while opponency is well established by both behavioral and physiological measures^131^, the red-green and blue-yellow axes of color appearance do not agree with the early precortical dimensions along which color is encoded (i.e. the L-M and S-LM mechanisms)^24^. This has suggested that the red-green and blue-yellow processes arise at later stages^132^ or are carried along different pathways^33,34^. However, there is also growing uncertainty around whether these canonical hue processes exist at all^118^. A clear neural signature for these perceptual processes has yet to be identified, and the special colors they predict (e.g. pure red-green or blue-yellow) do not appear special in many behavioral tasks^133–135^. Thus, the visual representation of color—at stages closer to our conscious percepts—is, in reality, very poorly understood. Measurements with color-anomalous observers can be used to test which aspects of these percepts can be equated across observers with fundamentally different receptor constraints, and this may in turn elucidate the basis for this equivalence. For example, neuroimaging studies have attempted to identify different stages of color coding and the cortical sites at which the neural representation of color begins to resemble the perceptual organization of color^136–138^. If these representations look different for routine and anomalous observers, yet their color reports agree, then we would need to look elsewhere to understand the nature of these reports. More generally, tests of plasticity and compensation in color vision deficiencies may reveal which types of measurements uncover intrinsic signals within our senses versus the cognitive strategies involved in their readout.

## Conclusion

While color vision deficiencies are often modeled as a reduced form of standard trichromatic color vision, many lines of evidence point to compensatory adjustments that can partially discount the consequences of the receptor loss or change. Understanding these adjustments—their number, nature, and limits—can shed important light on general principles of adaptation and plasticity in sensory systems and on the still poorly understood basis of human color experience.
